# Transcriptional Analyses Identify Genes That Modulate Bovine Macrophage Response to *Toxoplasma* Infection and Immune Stimulation

**DOI:** 10.3389/fcimb.2020.00437

**Published:** 2020-08-20

**Authors:** Anton Gossner, Musa A. Hassan

**Affiliations:** ^1^Division of Infection and Immunity, The Roslin Institute, The University of Edinburgh, Edinburgh, United Kingdom; ^2^Centre for Tropical Livestock Genetics and Health, The University of Edinburgh, Edinburgh, United Kingdom

**Keywords:** bovine toxoplasmosis, macrophages, RNA-sequencing, *Toxoplasma*, chemokine, Wnt/β-catenin-signaling

## Abstract

The obligate intracellular parasite, *Toxoplasma gondii*, is highly prevalent among livestock species. Although cattle are generally resistant to *Toxoplasma* strains circulating in Europe and North America, the underlying mechanisms are largely unknown. Here, we report that bovine bone marrow-derived macrophage (BMDM) pre-stimulated with interferon gamma (IFNγ) restricts intracellular *Toxoplasma* growth independently of nitric oxide. While *Toxoplasma* promoted the expression of genes associated with alternative macrophage activation and lipid metabolism, IFNγ abrogated parasite-induced transcriptional responses and promoted the expression of genes linked to the classical macrophage activation phenotype. Additionally, several chemokines, including *CCL22*, that are linked to parasite-induced activation of the *Wnt*/β-catenin signaling were highly expressed in *Toxoplasma*-exposed naïve BMDMs. A chemical *Wnt*/β-catenin signaling pathway antagonist (IWR-1-endo) significantly reduced intracellular parasite burden in naïve BMDMs, suggesting that *Toxoplasma* activates this pathway to evade bovine macrophage anti-parasitic responses. Congruently, intracellular burden of a mutant *Toxoplasma* strain (RHΔ*ASP5*) that does not secrete dense granule proteins into the host cell, which is an essential requirement for parasite-induced activation of the *Wnt*/β-catenin pathway, was significantly reduced in naïve BMDMs. However, both the *Wnt*/β-catenin antagonist and RH*ASP*Δ5 did not abolish parasite burden differences in naïve and IFNγ-stimulated BMDMs. Finally, we observed that parasites infecting IFNγ-stimulated BMDMs largely express genes associated with the slow dividing bradyzoite stage. Overall, this study provides novel insights into bovine macrophage transcriptional response to *Toxoplasma*. It establishes a foundation for a mechanistic analysis IFNγ-induced bovine anti-*Toxoplasma* responses and the counteracting *Toxoplasma* survival strategies.

## Introduction

*Toxoplasma* is a zoonotic protozoan parasite that infects virtually all warm-blooded vertebrates and is perhaps the most successful and widespread human pathogen. It is the leading cause of encephalitis and death in HIV/AIDS patients (Basavaraju, 2016) and is ranked 4th among foodborne parasites with the greatest global impact (FAO/WHO, 2014) and contributors to years lived with disability and disability-adjusted life years per million persons (Gkogka et al., 2011). *Toxoplasma* undergoes sexual reproduction exclusively in cats while asexual reproduction can occur in any intermediate host, including humans and farmed animals. Definitive-to-intermediate host parasite transmission occurs via ingestion of oocysts from infected cat feces, while transmission between intermediate hosts can occur through the consumption of contaminated food products or vertically from mother to fetus (Hill and Dubey, 2002). Although *Toxoplasma* infections in healthy individuals are mostly asymptomatic, severe disease or even death, often caused by damage to the brain or other organs, can occur in immunocompromised or congenitally infected individuals (Hill and Dubey, 2002). *Toxoplasma* typically establishes lifelong chronic infection in healthy intermediate hosts by encysting, after an initial phase of rapid intracellular proliferation and cell–cell spread, in the central nervous system and muscle tissues (Hill and Dubey, 2002). To do this, the parasite must carefully regulate immune activation and host cell anti-*Toxoplasma* effector mechanisms.

*Toxoplasma* invades host cells mostly through an active process powered by parasite-generated actin motor activity to form a specialized non-fusogenic parasitophorous vacuole (PV) (Fleckenstein et al., 2012) that helps the parasite avoid host cell immune surveillance (Morisaki et al., 1995). In phagocytic cells, such as macrophages, the parasite can also be taken up by phagocytosis and, sometimes the phagocytosed parasites can exit the phagosome to form a PV (Zhao et al., 2014). Besides hiding within the PV, the parasite also evades host cell immunity by sequentially discharging several effector proteins that modulate a variety of host immune and metabolic processes, including the inflammatory pathway (Hunter and Sibley, 2012). Generally, effective host anti-*Toxoplasma* responses are dependent on the production of interleukin (IL)-12 (Gazzinelli et al., 1994) by macrophages and dendritic cells (Gazzinelli et al., 1994). IL-12 in turn activates natural killer (NK) and T cells to secrete interferon gamma (IFNγ) (Gazzinelli et al., 1993, 1994), a pro-inflammatory cytokine that activates several anti-*Toxoplasma* effector mechanisms such as the interferon-regulated GTPases (IRGs) in mice (Zhao et al., 2009), reactive nitrogen/oxygen intermediates (Scharton-Kersten et al., 1997), tryptophan degradation and cell death in human cells (Pfefferkorn, 1984; Niedelman et al., 2013), and inflammasome activation (Cirelli et al., 2014). In return, *Toxoplasma* has evolved several mechanisms to counteract the IFNγ-induced host defenses, including the secretion of effectors proteins from specialized apical organelles that co-opt host transcription and signaling pathways to control host cell responsiveness to inflammatory signals (Jensen et al., 2011; Koshy et al., 2012; Bougdour et al., 2013; He et al., 2018).

Besides producing the IL12 that primes IFNγ secretion by NK and T cells, macrophages are also the preferred intracellular niche for the fast-dividing parasite stage (Jensen et al., 2011). Thus, the innate defenses triggered by monocytes/macrophages are key to toxoplasmosis pathogenesis in humans and rodents (Channon et al., 2000; Dunay and Sibley, 2010; Gregg et al., 2013; Tosh et al., 2016; Song et al., 2017). Besides killing microbes, including *Toxoplasma*, macrophages can also initiate adaptive immune responses (van de Vosse et al., 2009; Thi et al., 2012). Human and mouse models show that upon the engagement of surface signaling receptors or pattern recognition receptors (PRRs) such as, toll-like (TLRs), RIG-I-like (RLRs), and the cytosolic NOD-like (NLRs) receptors by conserved pathogen-associated molecular patterns (PAMPs) such as, lipopolysaccharide (LPS) or immune factors, including cytokines, macrophages assume different activation phenotypes. The most extreme classical [M1, M(IFNγ)] and the alternative [M2, M (IL-4)] phenotypes are separated by several intermediate activation states (Murray et al., 2014). The M1 phenotype, which can be induced by IFNγ, is highly microbicidal and characterized by the production of reactive oxygen and nitrogen intermediates such as nitric oxide (NO), and a range of pro-inflammatory cytokines and chemokines, such as Tumor necrosis factor (TNF) alpha (De Paoli et al., 2014; Murray et al., 2014). In contrast, the M2 phenotype, which is induced by IL-4 and IL-13 and is important for regulating inflammation, is characterized by the production of anti-inflammatory cytokines and growth factors (Sindrilaru and Scharffetter-Kochanek, 2013). The general hypothesis is that macrophage activation phenotypes, which are underpinned by discrete transcriptional programs (Hassan et al., 2015), provide a high degree of plasticity that is exploited by some intracellular pathogens, including *Toxoplasma* and *Mycobacteria*, to turn this potentially hostile host cell into a favorable replication niche (Price and Vance, 2014). Although effective host response to *Toxoplasma* require the induction of inflammation, characterized by classical macrophage activation, *Toxoplasma* promotes its survival in macrophages by secreting effector proteins to dampen inflammatory responses and favor alternative macrophage activation (Jensen et al., 2011). Indeed, *Toxoplasma* strain differences in virulence in mice is partly due to strain differences in inducing alternative macrophage activation (Jensen et al., 2011), while host differences in susceptibility to *Toxoplasma* is due in part to differences in macrophage activation phenotypes after infection (Jensen et al., 2011, 2013). Therefore, to devise strategies to improve the early defense against *Toxoplasma* and a variety of intracellular pathogens, it is important to understand the molecular mechanisms underpinning macrophage response to *Toxoplasma* and/or immune effector proteins, such as cytokines.

*Toxoplasma* is common in many species of livestock, including cattle. Compared to other livestock species, such as sheep and pigs, cattle are highly resistant to *Toxoplasma* and rarely transmit the parasite to other intermediate hosts (Dubey, 1986; Esteban-Redondo and Innes, 1997). Unlike sheep and pigs, natural *Toxoplasma* infection in cattle is mostly asymptomatic and does not appear to result in abortion. However, the molecular factors and mechanisms that modulate bovine-*Toxoplasma* interactions, which can be exploited to enhance resistance in other ruminants, are ambiguous. Although IFNγ is central to anti-*Toxoplasma* responses in virtually all vertebrates, the role of this cytokine, and the mechanism underpinning, bovine resistance to *Toxoplasma* is equivocal. In the present study we sought to determine the role of IFNγ, and host genes associated with effective bovine macrophage response to *Toxoplasma*. We performed RNA-sequencing on naïve or IFNγ-stimulated bovine bone marrow derived macrophages (BMDMs) that were either unexposed or exposed to a *Toxoplasma* strain that is highly virulent in laboratory inbred mice (RH) for 24 h. Analysis of the datasets provides novel insights into the *Toxoplasma*-induced transcriptional responses in naïve and pre-stimulated BMDMs. We report that IFNγ enhances bovine BMDMs anti-*Toxoplasma* responses and that, despite producing large amounts of nitric oxide, bovine macrophages restrict *Toxoplasma* independently of nitric oxide. *Toxoplasma* induces the Wnt/β-catenin signaling pathway and the expression of several anti-inflammatory chemokines and arginine metabolism in naïve BMDMs. On the other hand, to survive in IFNγ-primed BMDMs, the parasite expresses mostly genes that are associated with its slow dividing bradyzoite stage.

## Materials and Methods

### Parasites

The type I *Toxoplasma* strain (RH) engineered to express green fluorescent protein (GFP) and firefly luciferase has previously been described (Jensen et al., 2013). The RHΔ*ASP5* (Hammoudi et al., 2015) was a generous gift from Dr. Mohamed-Ali Hakimi (INSERM). All parasite strains were maintained by serial passage on confluent human foreskin fibroblast (HFF) monolayer.

### Primary Bone Marrow Derived Macrophages (BMDMs)

Marrow cells were flushed from the ribs of three (*n* = 3) ~2 year old calves using phosphate buffered saline (PBS, Invitrogen). The cells were centrifuged at 500 × g for 5 min at 4°C and re-suspended in red cell lysis buffer (Sigma) and incubated on ice for 5 min. Next, the cells were passed through a 70 μm cell strainer (BD Biosciences) and centrifuged at 500 × g for 5 min at 4°C. Cells from each calf were subsequently differentiated into macrophages in 10 cm non-tissue culture petri dishes (Corning) in RPMI 1640 (Sigma-Aldrich) supplemented with heat-inactivated 20% fetal bovine serum (FBS, Thermo Fisher Scientific), GlutaMAX (Thermo Fisher Scientific), penicillin/streptomycin (Thermo Fisher Scientific), and recombinant human CSF1 (10^4^ U/ml; a gift from Chiron, Emeryville, CA) for 10 days as previously described (Young et al., 2018).

### *In vitro* Measurements

BMDMs from each calf were detached using a cell scraper, washed, counted, and seeded separately in triplicates in 96-well plates at a density of 10^5^ cells/well. The BMDMs were left unstimulated (naïve) or stimulated with: recombinant bovine IFNγ (100 ng/mL), lipopolysaccharide (LPS) from *Salmonella enterica* serotype Minnesota Re 595 (Sigma-Aldrich, 100 ng/mL), or a combination of IFNγ (100 ng/mL) and LPS (100 ng/mL) with or without aminoguanidine (final concentration of 500 μM) and incubated at 37°C in 5% CO_2_. 24 h post-stimulation, cell free supernatants were collected for Griess reagent-based nitric oxide assay, as previously described (Young et al., 2018), and cell viability, using the CellTiter 96® Aqueous One Solution Cell Proliferation kit (Promega) according to the manufacturer's recommendations. Freshly lysed parasites (by sequential passage through 25G and 27G needles) were passed through a 5 μm filter to remove HFFs, counted, diluted in RPMI, and added to the BMDMs at a multiplicity of infection (MOI) 1 for 24 h before luciferase activity was measured using a luciferase assay kit (Promega) according to the manufacturer recommendations.

### RNA Sequencing and Analysis

2 × 10^6^ BMDMs from each calf were seeded in 6-well plates and left unstimulated or stimulated with IFNγ (100 ng/mL) for ~18 h at 37°C in 5% CO_2_. Next, the media was replaced with fresh media containing *Toxoplasma* at a MOI 1 and incubated further for ~18 h. Fresh media was added to the non-infected control BMDMs. At the end of the incubation period, total RNA was isolated from each BMDM sample using QIAzol® Lysis Reagent and miRNeasy Mini Kit (Qiagen) according to the manufacturer recommendations. RNA quality and integrity were assessed on the Agilent 2200 TapeStation using an Agilent RNA ScreenTape and quantified using a Qubit RNA BR Assay Kit and Fluorometer. RNA-seq libraries were generated and sequenced by Edinburgh Genomics. All libraries were prepared using the Illumina TruSeq Stranded library protocol for total RNA libraries (Part: 15031048, Revision E). Briefly, polyA-tail enrichment (Dynabeads mRNA Purification Kit; Invitrogen) was performed on the total RNA and the mRNA fragmented into 200–400 base-pairs, and reverse transcribed into cDNA before Illumina sequencing adapters were added to each end. Twelve barcoded libraries were multiplexed and sequenced on a single S2 sequencing lane on the Illumina NovaSeq 6000 machine to yield ~60 million 50 bp high quality strand-specific paired reads per sample. Reads were pseudoaligned to the Ensembl bovine genome (ARS-UCD1.2) using Kallisto v.0.46.0 (Bray et al., 2016) with 100 bootstraps to generate transcript-level expression estimates as transcripts per million (TPM) as previously described (Young et al., 2018). Gene-level differential expression analysis was performed in sleuth as previously described (Pimentel et al., 2017). TPM values derived from pseudoalignment of RNA-seq reads to the GT1 genome (ToxoDB.org) were used as input in sleuth to identify differentially expressed *Toxoplasma* genes.

### Quantitative Real-Time PCR

One microgram of total RNA from each BMDMs sample was reverse transcribed using Superscript III (Thermo Fisher Scientific), 10 mM dNTP mix (Thermo Fisher Scientific) and Oligo (dT)_12−18_ Primer (Thermo Fisher Scientific) according to the manufacturer's instructions. Gene-specific primers (Table 1) were designed using Primer-BLAST (Ye et al., 2012). All primers were commercially synthesized by Thermo Fischer Scientific. Quantitative real-time RT-PCR (qPCR) was performed in a Rotor-Gene Q real-time PCR cycler (Qiagen) using FastStart Universal SYBR Green Master (Rox) in final volume of 20 μL. The linearity and efficiency of qPCR amplification was determined for each primer pair using a standard curve generated by a serial dilution of cDNA pooled from all the samples. All reactions were performed in duplicate and “no template” controls included for each gene. Agarose gel electrophoresis was used to confirm product sizes and melt curve analysis confirmed specificity of amplification. Data were analyzed using the 2^−ΔΔ*Ct*^ method and statistical analyzed performed on the ΔCt values. qPCR was performed on parasite DNA as previously described (Li et al., 2000).

**Table 1:** Primer sets for qPCR validation of representative differentially expressed genes.

**Symbol**	**Primer 1 forward**	**Primer 1 reverse**	**Amplicon length**
*C1R*	GGTGCAGGATCAAGGACTGC	GTGTGCATCTTGTAGAAGGGCT	136
*ISG15*	GACCTGACGGTGAAGATGCTA	ATCTTCTGGGCGATGAACTGC	98
*PSMB8*	TGTCAATATGTACCACATGAAGGAG	CACCATCACTGACTGGCCTC	102
*SGK1*	GCCAAGGATGACTTTATGGAGA	AGGATCAAAGTGTCGCAGG	138
*IRF1*	AGGACATCATGAAGCTCTTGGA	GCTCCTCCTTGCAACTGAACT	129
*GBP5*	CCAGGAAAGGAATACAGGCTGA	TTCCATTGCTGTGAGAGCCAG	107
*RSAD2*	GTGGTTCCAGAAGTACGGTGA	CTTCTTTCCTTGACCACGGC	103
*CFB*	CTTGCAAAGGTGATTCTGGTGG	CGCTTGCAAACATCCACGAC	100
*GBP1*	CTCTCAAACTGCAGGAACAGTC	TGCTTTGGATAAGAGTGACCAG	175
*CXCL10*	TCCTCGAACACGGAAAGAGGCATA	AGCTGATATGGTGACTGGCTTGGT	164
*CCL22*	CGGGACTACATCCGTTACCC	CAGCACAGATCTCTCGGTCC	121
*CCl24*	GCAGGAGTGATCTTCACCACC	TAGCGGAGGCTTTCTTCTGC	115
*SDHA*	ACCTGATGCTTTGTGCTCTGC	CCTGGATGGGCTTGGAGTAA	126
*GAPDH*	GGTGATGCTGGTGCTGAGTA	TCATAAGTCCCTCCACGATG	265

## Results

### IFNγ Induces Restriction of *Toxoplasma* Growth in Bovine Macrophages Independently of Nitric Oxide

**Figure 1:**
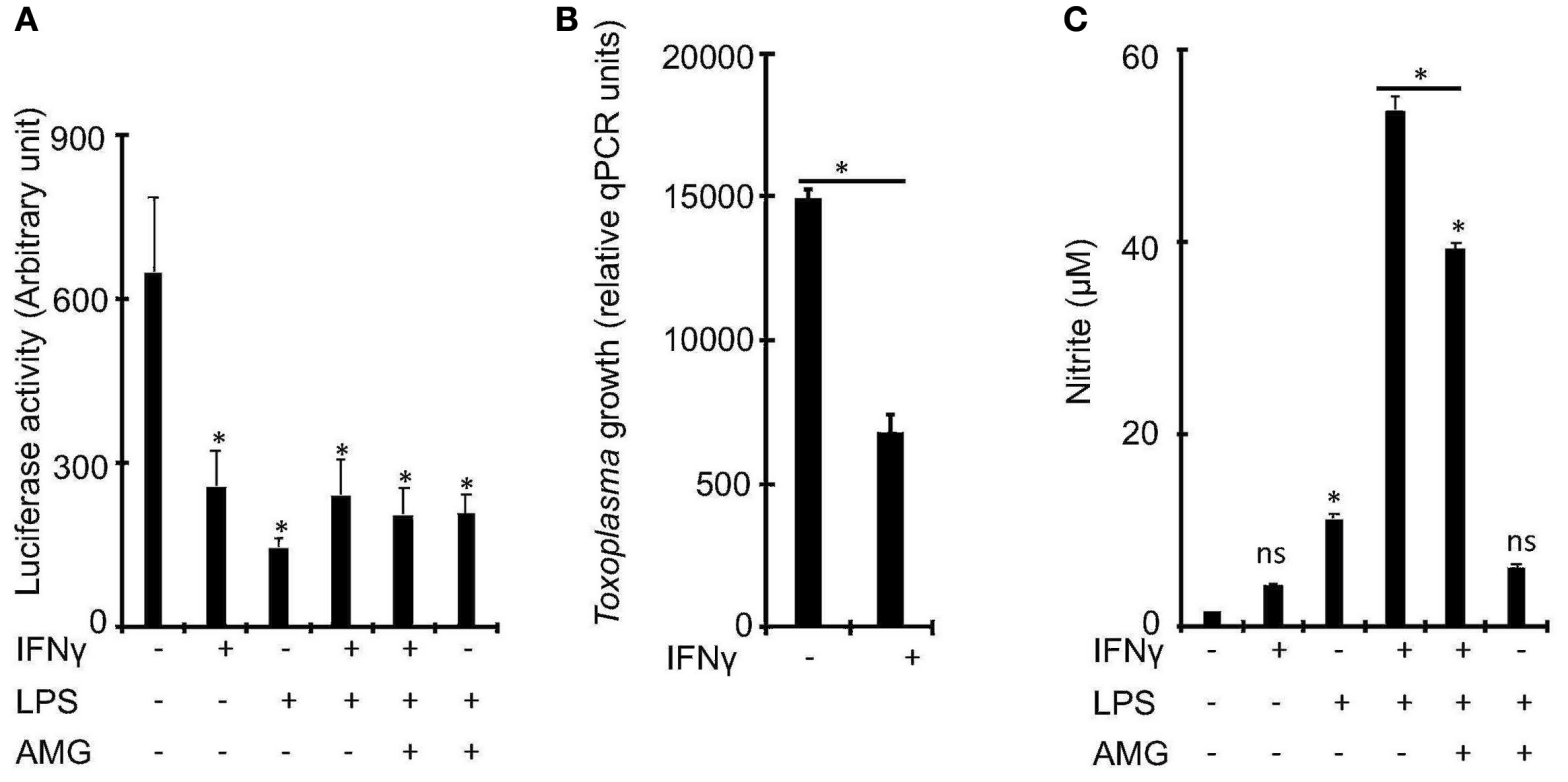
IFNγ restricts *Toxoplasma* growth in bovine bone marrow derived macrophages (BMDMs) independently of inducible nitric oxide. **(A)** Bovine BMDMs stimulated with IFNγ (100 ng/mL) and/or LPS (100 ng/mL) for ~18 h have reduced intracellular parasite burden, relative to control, which did not change with the addition of aminoguanidine (AG), a scavenger for reactive oxygen/nitrogen species, to the cell culture medium. **(B)** Reduced intracellular parasite burden in IFNγ-simulated BMDMs was confirmed using a quantitative real-time PCR (qPCR) on parasite DNA as previously described (Li et al., 2000). **(C)** Similar to murine, BMDMs, IFNγ alone, without a second stimulant, does not sufficiently induce NO in bovine BMDMs. Data are average value ±s.d. of three replicates. *P*-values of two-tailed unpaired Student's *t-*test; ^*^p < *0.05* and ns = not significant. Data are representative of three independent experiments.

To determine whether IFNγ is essential for the control of *Toxoplasma* growth in bovine macrophages, we exposed naïve or IFNγ-stimulated bovine bone marrow-derived macrophages (BMDMs) to a luciferase-expressing type I *Toxoplasma* strain (RH) for 24 h and measured luciferase activity, a proxy for parasite burden (Hassan et al., 2015). In murine macrophages, IFNγ is known to require a second stimulant, such as LPS or TNF, to effectively restrict *Toxoplasma* (Sibley et al., 1991; Hassan et al., 2015). Therefore, we included in our experiments, BMDMs that were pre-stimulated with IFNγ+LPS. As expected, there was reduced intracellular parasite burden in BMDMs pre-stimulated with IFNγ or IFNγ+LPS, relative to naïve BMDMs (Figure 1A). However, there were no significant differences in parasite burden between IFNγ- and IFNγ+LPS-stimulated BMDMs, suggesting that IFNγ alone can sufficiently induce anti-*Toxoplasma* activities in bovine BMDMs. A quantitative real-time polymerase chain reaction (qRT-PCR) analysis of parasite DNA (Li et al., 2000) confirmed the intracellular parasite burden differences between naïve and IFNγ-stimulated BMDMs (Figure 1B).

Previously, others and we reported that Nitric oxide (NO), a product of the inducible nitric oxide synthase (iNOS or NOS2)-catalyzed L-arginine metabolism (MacMicking et al., 1997), is a major IFNγ-induced effector against intracellular *Toxoplasma* growth in murine BMDMs (Hassan et al., 2015). In addition, bovine BMDMs stimulated with LPS and/or IFNγ are reported to produce significantly more NO than ovine or equine BMDMs (Denis et al., 2005; Young et al., 2018), which are susceptible to *Toxoplasma*. Thus, we postulated that NO inhibits *Toxoplasma* growth in bovine IFNγ-stimulated BMDMs. Consequently, we determined whether IFNγ alone can induce NO production in bovine BMDMs and whether the levels of the induced NO correlate with intracellular parasite burden in the pre-stimulated BMDMs. Unlike IFNγ, IFNγ+LPS induced a 34-fold increase in NO, relative to unstimulated BMDMs (Figure 1C). Thus, unlike murine BMDMs, NO is dispensable for IFNγ-induced *Toxoplasma* growth restriction in bovine BMDMs.

### *Toxoplasma* Induces a Robust Bovine Macrophage Transcriptional Response

**Figure 2:**
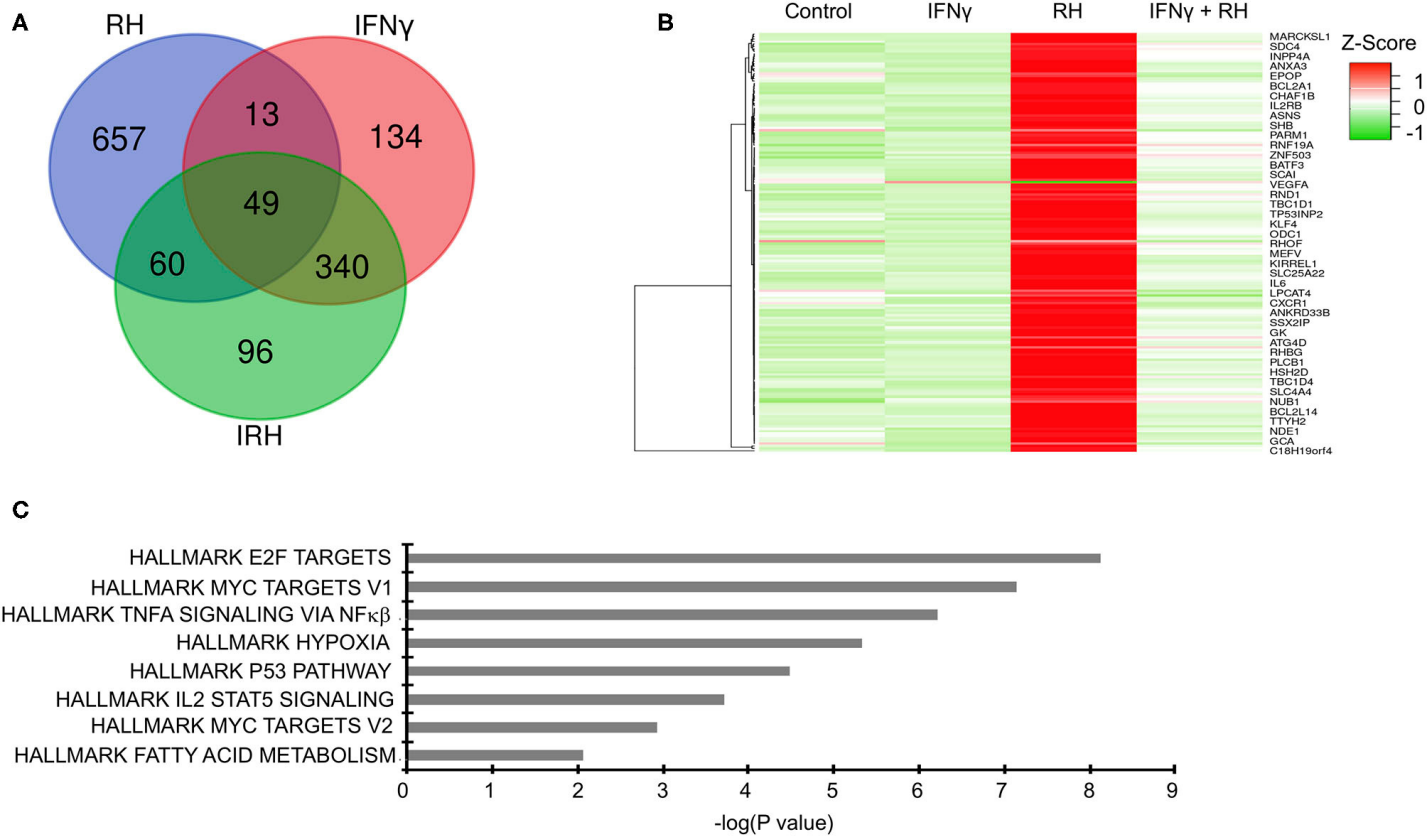
Distinct changes in gene expression underpin bovine macrophage response to IFNγ and/or *Toxoplasma*. **(A)** RNA-seq analysis of bovine BMDMs that were either left unstimulated and uninfected (Control), unstimulated and infected (RH), or stimulated with IFNγ (100 ng/mL) and uninfected (IFNγ) or stimulated with IFNγ and infected (IRH). Venn diagram representation of the number of dysregulated genes (≥2-fold; ≥10 uniquely aligned reads) relative to the control BMDMs. **(B)** Heatmap representation of genes dysregulated in RH BMDMs shows most of the stochastic changes in gene expression was due was due to *Toxoplasma*-induced increase in transcript abundance. **(C)** Enrichment analysis in the HALLMARK gene sets of the differentially expressed genes shows that *Toxoplasma* induced a unique gene sets, including the *Myc targets V1 and V2*.

To gain mechanistic insight into bovine innate immune response to *Toxoplasma*, we leveraged high throughput RNA-sequencing to profile the transcriptional landscape of bovine BMDMs that were left unstimulated (naïve) or pre-stimulated with IFNγ before being exposed to *Toxoplasma* for 24 h. Transcriptional analysis was also performed on uninfected controls. Downstream analysis was restricted to genes that were differentially expressed by more than 2-fold change and had at least 10 reads that uniquely aligned to the genome in at least two samples when compared the uninfected naïve BMDMs. In total, 1,349 unique genes were differential expressed in at least one condition, of which 887 were differentially expressed in a condition-specific manner (Figure 2A and Supplementary Table 1). Unlike IFNγ that interacts mainly with the IFNγ receptors to induce the expression of several IFNγ-specific genes (ISGs), *Toxoplasma* is likely to interact with many, yet to be defined, bovine BMDM pattern recognition receptors to induce bovine gene expression. Indeed, there were more differentially expressed genes that were unique to naïve BMDMs exposed to *Toxoplasma* (RH-specific) than in the uninfected IFNγ-stimulated BMDMs, (657 vs. 134, respectively; Hypergeometric *P-*value ≤ 0.05). Interestingly, only 96 genes were differentially expressed exclusively in IFNγ-stimulated BMDMs exposed to *Toxoplasma* (IRH-specific), suggesting that pre-stimulation with IFNγ abrogates a majority of *Toxoplasma*-induced transcriptional changes in the BMDMs (Figure 2A). The variable expression of most genes (579/657, ~88%), including immunoregulatory genes such as Krüppel-like factor 4 (*KLF4*) and Ornithine decarboxylase (*ODC1*), in the RH BMDMs was due to increased transcript abundance in the *Toxoplasma*-exposed BMDMs, rather than a downregulation of innately expressed genes, as revealed by hierarchical clustering (Figure 2B), suggesting that effective response to the parasite is inducible. Three hundred and forty genes, including several inflammatory cytokines such C-X-C motif chemokine ligand 10 (*CXCL10*) that were differentially expressed in unexposed IFNγ-stimulated BMDMs, remained dysregulated by a similar fold-change magnitude when the IFNγ-stimulated BMDMs were exposed to *Toxoplasma* (IRH), suggesting an inability by the parasite to overcome most of the IFNγ-induced transcriptional changes. Arginase 2 (*ARG2*) was among 13 genes that were highly expressed in RH but downregulated in IFNγ-stimulated BMDMs. Similarly, 60 genes, including the C-C motif chemokine 22 (*CCL22*), were upregulated in RH but downregulated by a difference of more than 2-fold change between in RH and IRH BMDMs.

To gain greater insights into the biological processes underpinning bovine BMDMs transcriptional response to *Toxoplasma*, and to determine whether the transcriptional changes in the different BMDM conditions was due to differences in the induction of distinct gene sets or the magnitude of induction of the same gene sets, we performed a pre-ranked gene set enrichment analysis (GSEA) (Subramanian et al., 2005) on the differentially expressed genes in IFNγ-stimulated, IRH, and RH BMDMs using the curated “HALLMARK” gene set database (Liberzon et al., 2015). Several “HALLMARK” gene sets, including the interferon gamma response, inflammatory response, and the regulation of cytokine production, were significantly (*FDR* ≤ 0.05) enriched in at least two BMDM conditions. Some gene sets, including hypoxia, which is not only a well-established *Toxoplasma*-induced host cell response, but also supports intracellular parasite growth (Wiley et al., 2010), were significantly enriched in RH but absent in IRH (Figure 2C). Thus, infection of naïve and IFNγ-stimulated BMDMs modulated by differences in the induction of unique and common gene sets.

### IFNγ Reverses *Toxoplasma*-Induced Dysregulation of Genes Linked to Inflammatory and Metabolic Pathways to Restrict Parasite Replication in Bovine Macrophages

**Figure 3:**
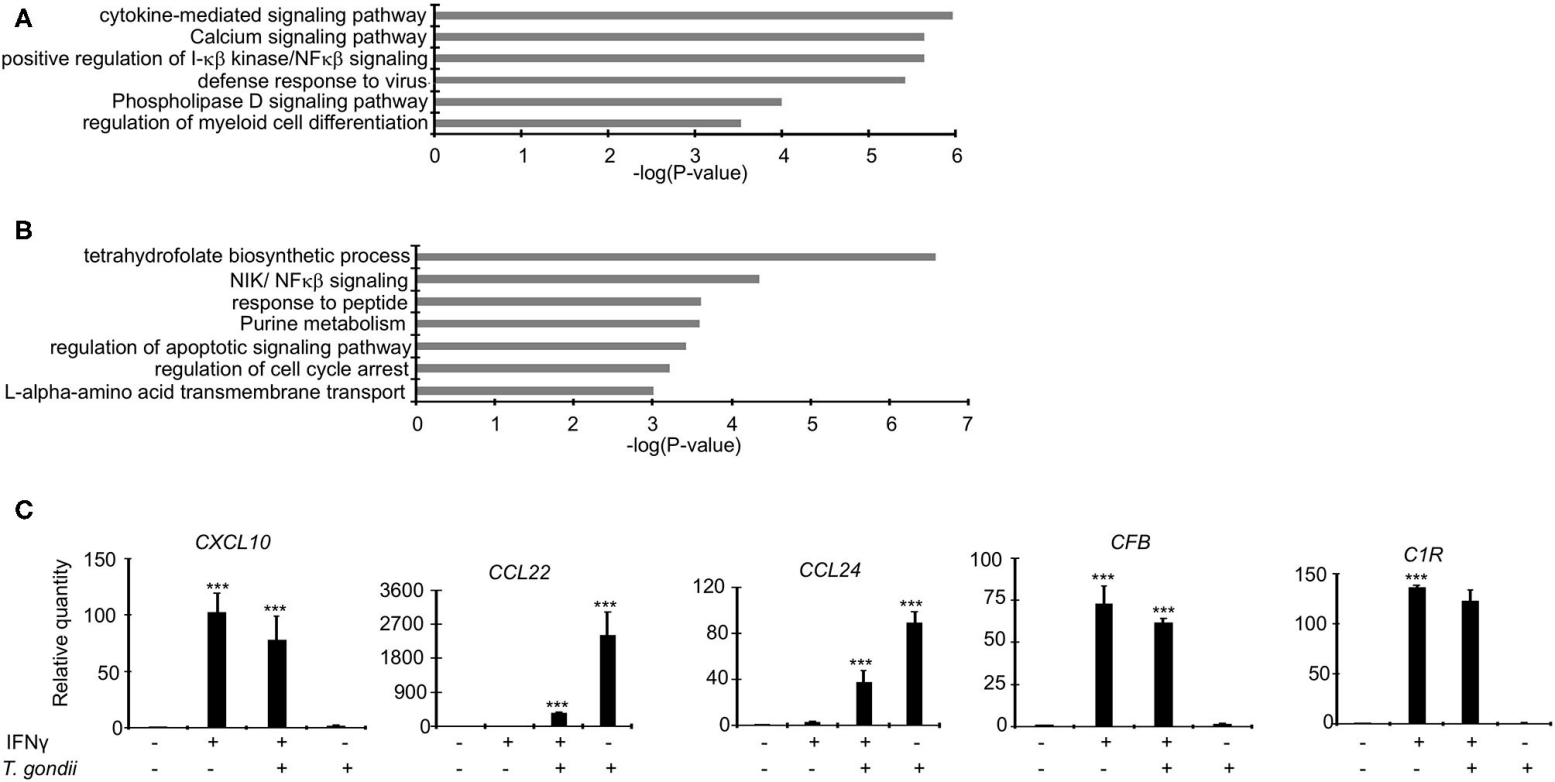
Dysregulated genes in RH and IRH are enriched in unique and common functional terms. Enrichment analysis in functional annotation and gene ontology (GO) analysis of the differentially expressed genes using metascape show that enrichment for unique functional terms in **(A)** Genes upregulated and/or unchanged IRH BMDMs, and **(B)** Genes upregulated in RH but not IRH BMDMs. **(C)** A subset of differentially expressed genes were validated in quantitative real-time polymerase chain reaction (qPCR) in naïve infected (RH), IFNγ-stimulated and infected (IRH), and IFNγ-stimulated BMDMs. Data are average value ±s.d. of three replicates. *P*-values of two-tailed unpaired Student's *t-*test; ^***^p < *0.001*. Data are representative of two independent experiments.

As demonstrated above, *Toxoplasma* significantly dysregulate several bovine genes, potentially to support its intracellular survival. We reasoned that since IFNγ-stimulated BMDMs are refractory to the parasite, *Toxoplasma*-induced genes that support the intracellular parasite lifestyle are likely to be targeted and reversed when BMDMs are pre-stimulated with IFNγ prior to infection. Several inflammatory genes were dysregulated in IRH but not in RH BMDMs. For example, *CXCL9, 10*, and *11*, were 22-, 10-, and 12-fold, respectively, upregulated in IRH, but not dysregulated in RH, BMDMs. Markers for the *Toxoplasma*-refractory classically activated macrophages, such as *CD180, CD74*, and *CD1D* were highly expressed in IRH but not in RH BMDMs. Similarly, c-type lectins including *CLEC6A*, which are associated with classically activated macrophages (Jensen et al., 2011; Murray et al., 2014), were upregulated in IRH BMDMs. Conversely, calcium signaling genes that are reported to play a significant role in intracellular parasite survival, such as *CAMK2G*, and histone deacetylase (*HDAC*) 5, and 7, were downregulated in RH, but remained unchanged in IRH BMDMs. A functional analysis on genes upregulated or unchanged in IRH BMDMs revealed an enrichment for, among others, calcium signaling pathway, phospholipase D signaling, defense response to virus, and positive regulation of I-κβ kinase/NFκβ signaling (Figure 3A).

Genes implicated in chemotaxis or anti-inflammatory responses were differentially expressed by a margin of more than 2-fold change between RH and IRH BMDMs. For example, chemotaxis-related *CCL22* and *CCL24* chemokines were upregulated by 20- and 12-fold in RH but upregulated by 14- and 8-fold in IRH BMDMs. Similarly, the chemokine receptor (*CCR7)* was upregulated by 26-fold in RH but not differentially expressed in IRH BMDMs. The anti-inflammatory-related suppressor of cytokine signaling (*SOCS*) 3, and TNF receptor-associated factor 1 (*TRAF1*) were also upregulated in RH but downregulated in IRH BMDMs. Mitogen-activated protein kinases (MAPK), including *MAP3K14* and *MAP4K1*, which induce non-canonical (Jin et al., 2014) and suppresses canonical (Brenner et al., 2005) NF-κB signaling pathways, respectively, were upregulated in RH but downregulated in IRH BMDMs. Studies in model animals, including mice, have shown that *Toxoplasma* induces alternative macrophage activation to favor its intracellular lifestyle (Melo et al., 2011). Arginine metabolism, via arginase, is key to alternative macrophage activation. Congruently, Arginase 2 (*ARG2*) was 4-fold upregulated in RH but virtually not expressed in IRH BMDMs. Additional genes associated with alternative macrophage activation, such as *KLF4* and *IL34* were upregulated in RH but downregulated in IRH BMDMs. The expression of protein arginine methyltransferase 5 (*PRMT5*), which is necessary in c-MYC-mediated alternative macrophage differentiation, was slightly increased in RH BMDMs. Similarly, genes encoding glucose transporters were upregulated in RH but not IRH BMDMs. Solute carriers (*SLC*), including *SLC2A1* and *SLC2A3* that encode glucose transporters, were downregulated in IRH but upregulated in RH BMDMs. Other solute carriers, including *SLC7A1* and *SLC7A5* that function in the L-arginine transport pathway, and *ODC1*, the rate limiting enzyme in polyamine biosynthesis and a negative regulator of macrophage inflammation (Hardbower et al., 2017), were upregulated in RH BMDMs. *Toxoplasma* reportedly rely on host lipid droplets, which increase in abundance in *Toxoplasma*-infected cells, to sustain rapid intracellular replication (Gomes et al., 2014). The expression of pentraxin 3 (*PTX3*), which is induced by lipid accumulation (Liu et al., 2014), was increased 8-fold in RH but unexpressed in IRH BMDMs. Similarly, the expression of genes involved in fatty acid elongation, such as *ELOVL6*, were upregulated in RH but not expressed in IRH BMDMs. Genes upregulated in RH but not IRH BMDMs were significantly (*FDR* ≤ 0.05) linked to purine metabolism, NIK/NFκβ (non-canonical NFκβ) signaling, and regulation of apoptotic signaling pathway (Figure 3B), all of which are expected to play a role in regulating *Toxoplasma*'s intracellular fate (Yamamoto et al., 2011; Blume and Seeber, 2018). Quantitative RT-PCR (qPCR) confirmed the differential expression of genes, including *CXCL10, CCL22*, and *CCL24* that are reportedly upregulated in *Toxoplasma*-infected murine macrophages (Melo et al., 2013), in the different macrophage conditions (Figure 3C and Supplementary Figure 1). Thus, IFNγ targets both the host cell inflammatory and metabolic pathways to revoke intracellular parasite replication.

### IFNγ Abrogates *Toxoplasma*-Induced *Wnt*/β-Catenin Signaling in Bovine Macrophages

Recent studies have documented *Toxoplasma-*induced expression of several chemokines, including *CCL17, CCL22*, and *CCL24* (He et al., 2018; Majumdar et al., 2019) via the activation of the *Wnt*/β-catenin signaling pathway (He et al., 2018). *Wnt/*β-catenin-signaling has an important role in various cellular processes, including proliferation (Reya et al., 2003) and immunity (Staal et al., 2008). The variable expression of *CCL22* and *CCL24*, in RH and IRH BMDMs, prompted us to investigate the expression of *Wnt*-target genes to establish a role of *Wnt/*β-catenin signaling in bovine BMDM response to *Toxoplasma*. However, apart from the downregulation of delta like canonical Notch ligand 1 (*DLL1*) in IFNγ-stimulated BMDMs, none of the canonical *Wnt*-target genes were differentially expressed in RH and/or IRH BMDMs.

**Figure 4:**
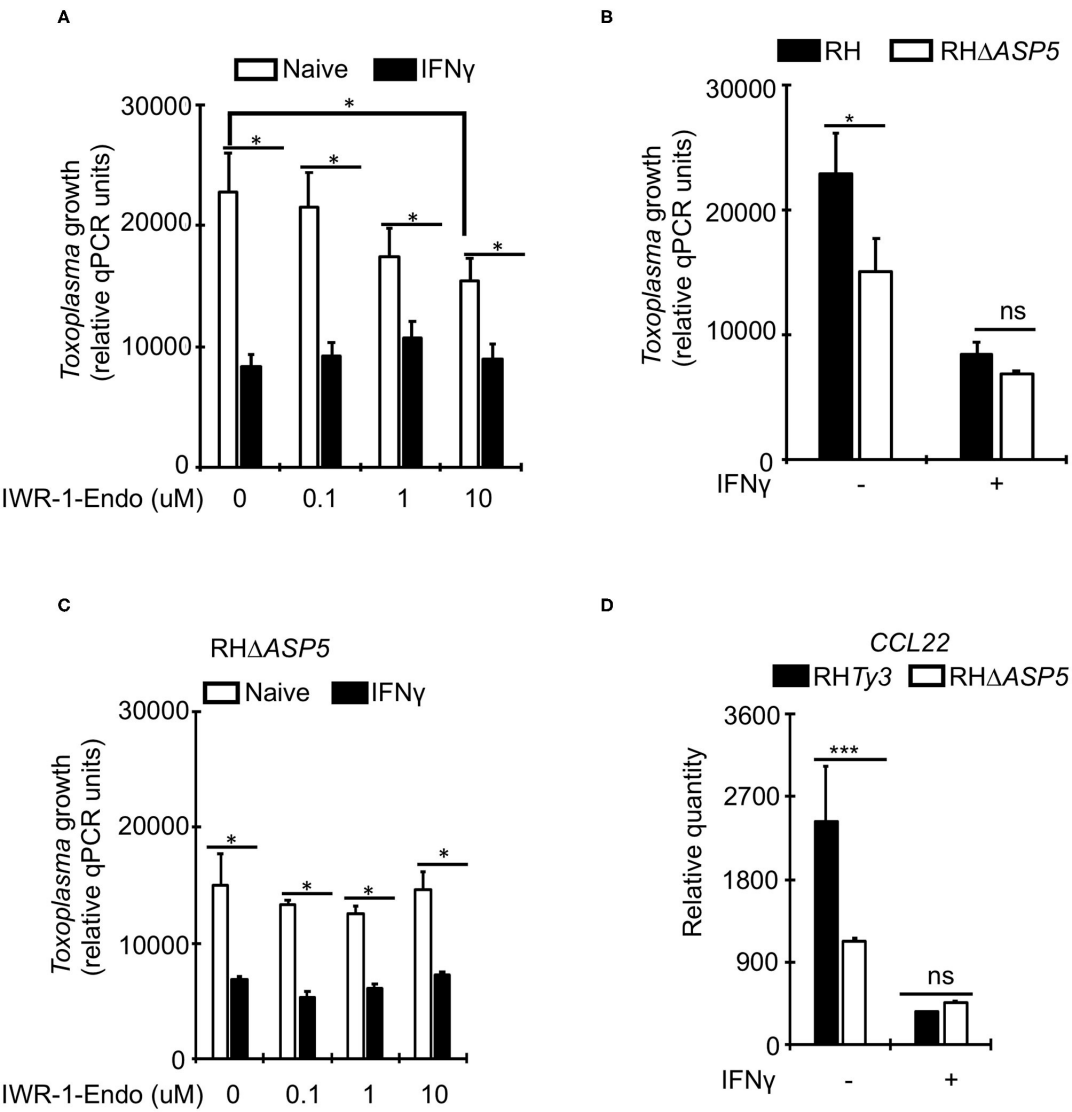
A role for *Wnt*/β-catenin signaling in the IFNγ-induced response to *Toxoplasma* in bovine macrophages. **(A)** Naïve (white bars) or IFNγ-stimulated (black bars) were cultured overnight with or without increasing concentrations of the *Wnt*/β-catenin signaling antagonist IWR-1-endo and infected with a parental wildtype (RHTy3) *Toxoplasma* strains. IWR-1-endo did not abolish differences in intracellular parasite burden between naïve and IFNγ-stimulated BMDMs. **(B)** The growth of the knockout RHΔ*ASP5* parasite strain was significantly inhibited in naïve BMDMs infected, relative to the wildtype. RHΔ*ASP5* parasites do not secrete most dense granule proteins, including GRA18, beyond the parasitophorous vacuole membrane. **(C)** Unlike wildtype parasites, IWR-1-endo did not significantly alter parasite burden in naïve or IFNγ-stimulated BMDMs infected with RHΔ*ASP5*. **(D)** The parental RHTy3 strain induced significantly higher expression of *CCL22* in naïve BMDMs when compared to the knockout RHΔ*ASP5* strain. Data are average value ±s.d. of three replicates. *P*-values of two-tailed unpaired Student's *t-*test; *p < *0.05*, ***p < 0.001 and ns = not significant. Data are representative of three independent experiments.

Unlike *Toxoplasma*, IFNγ is known to suppress *Wnt*/β-catenin signaling (Nava et al., 2014; Bai et al., 2017). To determine whether *Toxoplasma* growth restriction in IFNγ-stimulated bovine BMDMs is linked to *Wnt*/β-catenin signaling, we evaluated parasite burden in naïve or IFNγ-stimulated BMDMs cultured in the presence or absence of a *Wnt*/β-catenin antagonist (IWR-1-endo). IWR-1-endo reduced parasite burden in naïve BMDMs in a dose-dependent manner without abolishing the differences in parasite burden between naïve and IFNγ-stimulated BMDMs (Figure 4A). *Toxoplasma-*induced accumulation of β-catenin in the host cell nucleus, and the dysregulation of *CCL22* is reportedly induced by a dense granule (GRA) protein (GRA18) that is secreted beyond the parasites' parasitophorous vacuole membrane (PVM) into the host cell during infection (He et al., 2018). Thus, to overcome the potential effect of IWR-1-endo on unrelated host cell functions and establish a direct link between infection and *Wnt*/β-catenin activation, we infected naïve or IFNγ-stimulated BMDMs with a mutant *Toxoplasma* strain (RHΔ*ASP5*) and quantified parasite burden using qPCR. The RHΔ*ASP5* mutant strain lacks the ASP5 enzyme that is essential for the processing of GRA proteins, including GRA18, that are destined for secretion into the host cell (Coffey et al., 2015; Hammoudi et al., 2015; Curt-Varesano et al., 2016; He et al., 2018). We reasoned that if GRAs that are secreted in the host cell interact with β-catenin to induce chemokine secretion and support intracellular parasite survival and replication in bovine BMDMs, then the RHΔ*ASP5* strain should not be able to replicate efficiently in naïve BMDMs. Indeed, parasite burden was significantly reduced in naïve BMDMs infected with RHΔ*ASP5*, relative to the wildtype (Figure 4B). Unlike wildtype parasites, IWR-1-endo did not significantly alter parasite burden in naïve or IFNγ-stimulated BMDMs infected with RHΔ*ASP5* (Figure 4C). Neither did IWR-1-endo abrogate intracellular RHΔ*ASP5* burden differences between naïve and IFNγ-stimulated BMDMs. Finally, when infected with RHΔ*ASP5*, but not the wildtype parental strain, naïve BMDMs exhibited high abundance for *CCL22* transcripts (Figure 4D). Together, these results indicate a role for *Wnt*/β-catenin signaling and parasite secreted GRAs that translocate to the host cell in bovine BMDMs anti-*Toxoplasma* responses.

### Parasites in IFNγ-Stimulated Bovine Macrophages Transcribe Mostly Bradyzoite-Related Genes

**Figure 5:**
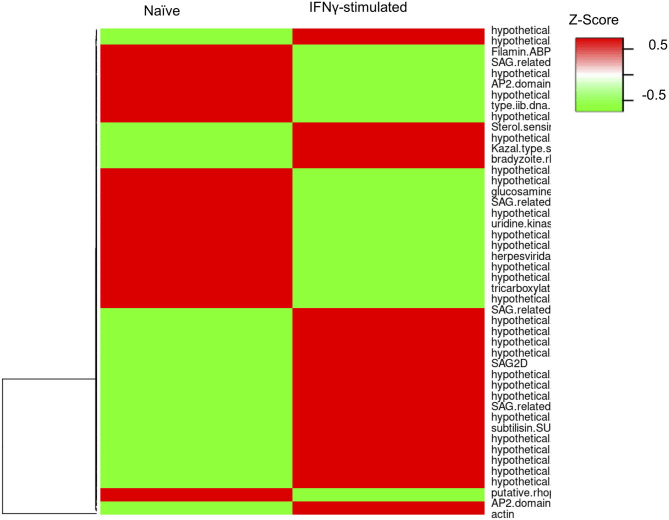
*Toxoplasma* gene expression varies in naïve and stimulate bovine macrophage. Heatmap representation of the differentially expressed genes (≥3-fold; ≥10 uniquely aligned reads in at least two samples) between parasites in naïve (RH) and IFNγ-stimulated (IRH) BMDMs.

To evaluate whether the infection of bovine BMDMs is underpinned by distinct parasite expression signatures, we used the RNA-sequencing reads uniquely aligning to the parasite genome (GT1 v.46) to evaluate stochastic changes in *Toxoplasma* transcript abundance in naïve and IFNγ-stimulated BMDMs. We focused our analysis on genes that were modulated by more than 3-fold change and had more than 10 uniquely aligned RNA-sequencing reads in at least two samples when comparing parasites infecting naïve and IFNγ-stimulated BMDMs. One hundred and eighty one genes were differentially expressed, of which 94 corresponded to genes upregulated in IFNγ-stimulated BMDMs (Figure 5 and Supplementary Table 1). Parasite genes that were highly expressed when the parasite is in IFNγ-stimulated BMDMs included DnaK-tetratricopeptide repeat (*DnaK-TPR*) and Cyclic AMP-Dependent Protein Kinase Subunit 3 (*cAMPK3*), which are associated with or involved in stress-induced *Toxoplasma* stage conversion from the fast dividing tachyzoite to the semi-dominant bradyzoite parasite stage (Ueno et al., 2011; Sugi et al., 2016). Thus, consistent with the well-defined immune pressure-induced parasite stage conversion, *Toxoplasma* potentially responds to the IFNγ-induced immune pressure by slowing down its rate of replication.

## Discussion

The outcome of *Toxoplasma* infections in cattle is fundamentally different from that of rodents, small ruminants, and pigs: clinical disease in naturally infected cattle are rare and many large-scale studies have failed to detect viable parasites in bovine tissues (Stelzer et al., 2019), suggesting that, like humans, cattle are largely an end-stage host for the parasite. However, the mechanisms underlying bovine resistance to *Toxoplasma* are largely unknown. In this study, we performed high throughput functional genomics and parasitological assays to investigate the molecular factors that modulate *Toxoplasma* interactions with bovine bone marrow-derived macrophages (BMDMs). Similar to observations in rodent and human macrophages, interferon gamma (IFNγ) significantly enhanced intracellular parasite growth restriction in bovine BMDMs. Interestingly, despite the ability to restrict intracellular parasite growth, IFNγ on its own was not able to induce nitric oxide (NO) secretion in bovine BMDMs, indicating that, unlike murine BMDMs, IFNγ-induced toxoplasmastatic activities in bovine BMDMs are independent of NO. Additionally, we found that secreted parasite dense granule proteins potentially induce the expression of chemokines, including *CCL22*, to enhance parasite growth in naïve bovine BMDMs.

Understanding the mechanisms of innate recognition and response to *Toxoplasma* in mice has been invaluable in advancing knowledge on response to *Toxoplasma* in other vertebrates, including cattle. The IFNγ-induced murine anti-*Toxoplasma* effectors, which are largely dependent on the activation of immunity-related GTPases (IRGs) and guanylate binding proteins (GBPs), are well-characterized. However, cattle, like humans, lack functional toll-like receptor (TLR) 11 and 12 (O'Neill et al., 2013) that facilitate the recognition of *Toxoplasma*-derived pathogen-associated molecular patterns (PAMPs) in murine cells (Melo et al., 2010). Although bovine BMDMs express a functional TLR5, unlike murine TLR5, it does not recognize *Toxoplasma* antigens (Tombácz et al., 2018). Thus, the mechanisms by which bovine cells recognize and respond to *Toxoplasma* are equivocal and potentially different from those defined in the murine system. Previous studies, including our own (Jensen et al., 2013; Hassan et al., 2015), show that reactive oxygen and nitrogen species, including NO, are central to *Toxoplasma* growth inhibition in murine macrophages synergistically activated with IFNγ and LPS or TNF. Bovine macrophages are known to secrete significantly more NO in response to LPS than sheep and pigs, which are susceptible to *Toxoplasma*, LPS (Jungi et al., 1996; Young et al., 2018), suggesting that species-specific differences in susceptibility to *Toxoplasma* is due to differences in NO production. Although parasite growth was significantly restricted in stimulating bovine BMDMs with IFNγ-stimulated bovine BMDMs, IFNγ, on it's own, did not induce NO in bovine BMDMs, which is consistent with previous observations (Denis et al., 2005; Young et al., 2018; Imrie and Williams, 2019). In fact, intracellular parasite burden in IFNγ- and IFNγ+LPS-stimulated BMDMs were similar, despite the latter secreting significantly more NO. Combined, these results suggest that NO is dispensable in IFNγ-induced bovine BMDM toxoplasmastatic activities. A similar non-essential role for NO in macrophage response to *Toxoplasma* has previously been observed in human macrophages. However, human macrophages are distinct from bovine BMDMs, since NO cannot be induced by conventional activating regimes *in vitro* in human macrophages (Schneemann and Schoedon, 2002).

Recent studies have documented a role for *Wnt*/β-catenin signaling in host responses to *Toxoplasma*. Enhanced intracellular *Toxoplasma* growth was found to co-occur with increased β-catenin (*CTNNB1*) gene expression in human cells (Majumdar et al., 2019), while a parasite secreted GRA protein is reported to stabilize β-catenin in murine BMDMs leading to an increased expression of chemokines such as *CCL22* (He et al., 2018; Majumdar et al., 2019). Consistent with IFNγ-induced inhibition of the *Wnt*/β-catenin signaling pathway (Nava et al., 2014; Bai et al., 2017), we observed a downregulation of several *Wnt*/β-catenin signaling-associated genes, including *CCL17, CCL22*, and *CCL24* (He et al., 2018), in IFNγ-stimulated BMDMs. Congruently, a *Wnt*/β-catenin signaling chemical inhibitor (IWR-1-endo) reduced intracellular parasite burden in naïve BMDMs in a dose-dependent manner. However, IWR-1-endo did not abolish intracellular parasite burden differences between naïve and IFNγ-stimulated BMDMs, probably because IFNγ suppresses *Wnt*/β-catenin signaling to a level below which further inhibition produces no discernible effect. Considering that we used a single IFNγ concentration (100 ng/mL), it is plausible that IWR-1-endo would have an effect at lower IFNγ concentrations. A more plausible explanation for the lack of IWR-1-endo efficacy in IFNγ-stimulated cells is that besides the *Wnt*/β-catenin signaling, IFNγ induces additional effector mechanisms against the parasite. Although GRA18 is reported to induce β-catenin accumulation in the host cell nucleus, this was not discernible when GRA18 was expressed at physiological levels (He et al., 2018), which may partly explain the lack of β-catenin nuclear accumulation in the present study. Nevertheless, intracellular parasite burden in bovine BMDMs infected with a mutant *Toxoplasma* strain that does not secrete GRA18 into the host cell cytoplasm (RHΔ*ASP5*) (He et al., 2018), was significantly different from cells infected the wildtype parasites. Similar to IWR-1-endo, infection with RHΔ*ASP5* did not abolish intracellular parasite burden differences between naïve and IFNγ-stimulated BMDMs.

The mechanisms regulating the impact of *Wnt*/β-catenin on intracellular *Toxoplasma* replication are largely equivocal. Recent studies indicate that β-catenin alters intracellular parasite growth dynamics by interacting with indoleamine 2,3-dioxygenase 1 (IDO1): *IDO1* promoter activity is supported by β-catenin. IDO1 is an IFNγ-inducible protein that degrades tryptophan to kynurenine and is known to impede *Toxoplasma* growth in human fibroblasts (Pfefferkorn, 1984), epithelial, and endothelial cells (MacKenzie et al., 2007) *in vitro*. Because IDO1 degrades tryptophan, which supports intracellular *Toxoplasma* replication, increased IDO1 promoter activity in the presence of β-catenin may appear contradictory. However, IDO1 transcript and protein levels are often discordant in *Toxoplasma*-infected cells: *Toxoplasma* promotes the degradation of IDO1 protein (Majumdar et al., 2019). Although we cannot conclude, from this study, that tryptophan plays an essential role in bovine BMDM response to *Toxoplasma, IDO1* was significantly downregulated in naïve BMDMs exposed to *Toxoplasma*. In the absence of IDO1, tryptophan is catabolized to melatonin, which scavenges reactive oxygen species (ROS) and promotes cell survival (Dolado and Nebreda, 2008), both of which are beneficial for intracellular *Toxoplasma* survival. However, considering that NO and ROS are dispensable for *Toxoplasma* growth restriction in bovine BMDMs, scavenging of ROS is unlikely to be a viable mechanism by which β-catenin can affect parasite replication. Rather, it is plausible that melatonin promotes cell survival, which in turn promotes intracellular parasite survival. Indeed, we did not observe significant changes in cell viability in *Toxoplasma*-infected, relative to uninfected, BMDMs. Alternatively, *Wnt*/β-catenin-mediated anti-*Toxoplasma* mechanisms may involve the induction of alternative macrophage activation, which is supportive of intracellular *Toxoplasma* survival and growth (Melo et al., 2011). Besides, the expression of *Wnt*/β-catenin-associated chemokines, we observed increased expression of genes associated with alternatively activated or foamy macrophages, such as *KLF4, ARG2, ODC1*, and *PTX3*, in naïve, but not IFNγ-stimulated, BMDMs. Combined, with the increased expression of glucose transporters in naïve BMDMs, which is reversed in IFNγ-stimulated BMDMs, we conclude that *Toxoplasma* exploits the bovine BMDMs metabolic pathways to enhance intracellular survival and replication. It is worth noting that we have tested only one clonal parasite strain (RH) at a single MOI and that different *Toxoplasma* strains, including the genetically distinct atypical strains such as GUYDOS, and higher MOIs may induce significantly different responses in the bovine BMDMs.

## Data Availability Statement

The original contributions presented in the study are publicly available. This data can be found here: https://www.ncbi.nlm.nih.gov/ PRJNA646376.

## Ethics Statement

The animal study was reviewed and approved by the Protocols and Ethics Committees of The Roslin Institute, The University of Edinburgh, and the Royal (Dick) School of Veterinary Medicine. In accordance with the United Kingdom Animal (Scientific Procedures) Act 1986, this study did not require a Home Office project license as no regulated procedures were carried out.

## Author Contributions

MH conceived and designed the experiments and wrote the manuscript. MH and AG performed the experiments and analyzed the data. All authors contributed to the article and approved the submitted version.

## Conflict of Interest

The authors declare that the research was conducted in the absence of any commercial or financial relationships that could be construed as a potential conflict of interest.

## Supplementary Material

Supplementary Figure 1 Quantitative real-time polymerase chain reaction (qPCR) of some differentially expressed genes in naïve infected (RH; black bars), IFNγ-stimulated and infected (IRH; blue bars), and IFNγ-stimulated (red bars), unstimulated-uninfected control (open bars) BMDMs. Data are average value ± s.d. of three replicates. Data are representative of two independent experiments.
