# Supplementary figures and images for: Transcriptional Analyses Identify Genes That Modulate Bovine Macrophage Response to Toxoplasma Infection and Immune Stimulation
Source: Front Cell Infect Microbiol. 2020 Aug 20;10:437. doi: 10.3389/fcimb.2020.00437 (PMC7508302; doi:10.3389/fcimb.2020.00437)

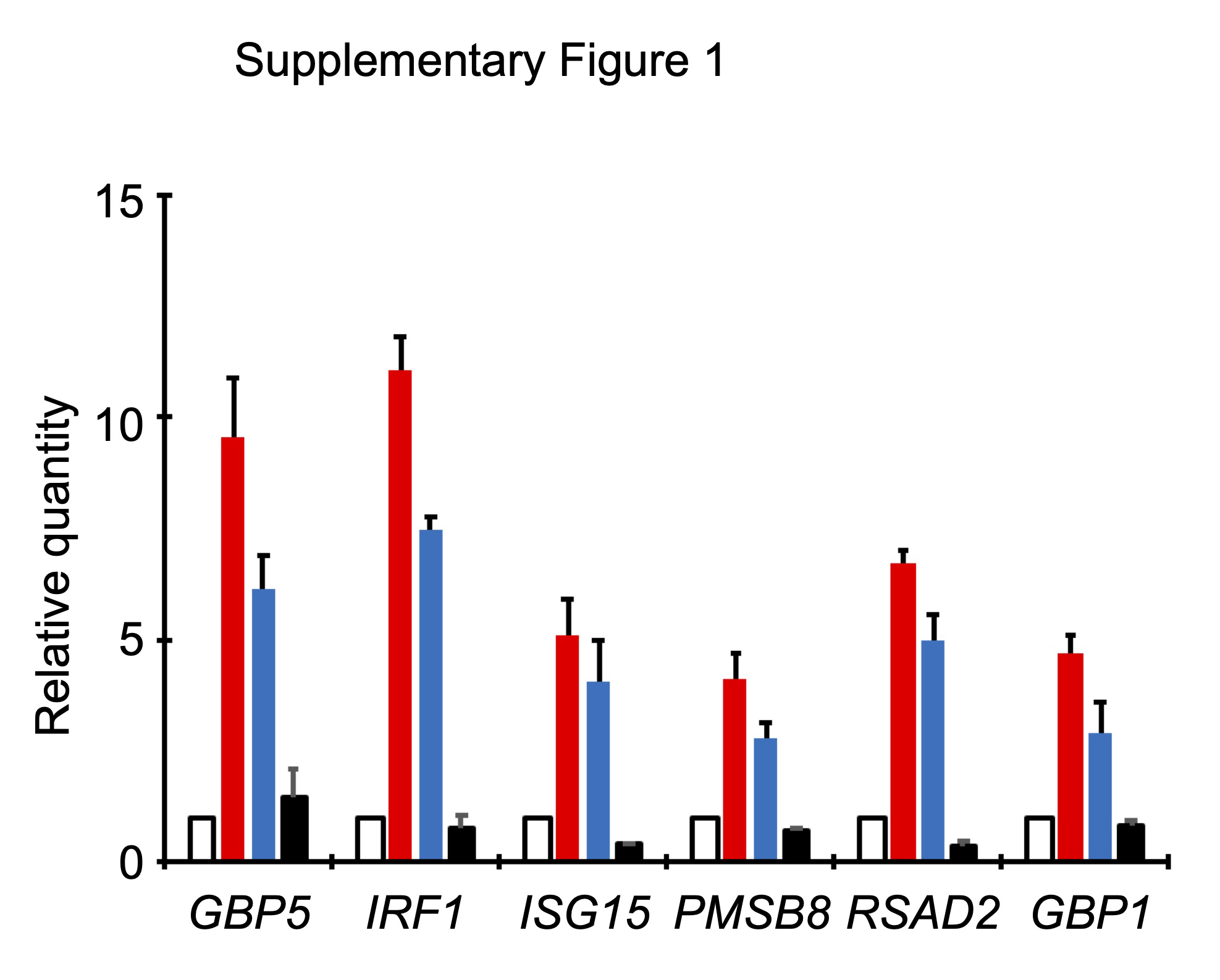

Supplement: Supplementary Figure 1 — Quantitative real-time polymerase chain reaction (qPCR) of some differentially expressed genes in naïve infected (RH; black bars), IFNγ-stimulated and infected (IRH; blue bars), and IFNγ-stimulated (red bars), unstimulated-uninfected control (open bars) BMDMs. Data are average value ± s.d. of three replicates. Data are representative of two independent experiments. [file Image_1.TIFF]
